# Plant-Derived Vesicle-like Nanoparticles: Pioneering Sustainable and Effective Approaches for Tissue Repair and Regeneration

**DOI:** 10.3390/biom15081055

**Published:** 2025-07-22

**Authors:** Qinjing Wang, Zhijie Huang, Jiming Guo, Weixing Chen, Min Wang, Yue Ming, Hongyu Liu, Mingshu Huang, Yisheng Huang, Zhengming Tang, Bo Jia

**Affiliations:** Stomatological Hospital, School of Stomatology, Southern Medical University, Guangzhou 510261, China; wangqinjing1126@163.com (Q.W.); zhijie@smu.edu.cn (Z.H.); w1786736226@163.com (J.G.); weix__chen@163.com (W.C.); 15989263674@163.com (M.W.); ming15342664475@163.com (Y.M.); lhy007123456@163.com (H.L.); hms6124357@163.com (M.H.); hysxuexi@163.com (Y.H.); tangzmet@163.com (Z.T.)

**Keywords:** plant-derived vesicle-like nanoparticles, tissue repair, regenerative medicine, nanotherapeutics, drug delivery

## Abstract

Plant-derived vesicle-like nanoparticles (PDVLNs) are bioactive nanovesicles secreted by plant cells, emerging as a novel therapeutic tool for tissue repair and regeneration due to their low immunogenicity, intrinsic bioactivity, and potential as drug delivery carriers. This review examines PDVLNs’ biogenesis mechanisms, isolation techniques, and compositional diversity, emphasizing their roles in promoting essential regenerative processes—cell proliferation, differentiation, migration, immune modulation, and angiogenesis. We explore their therapeutic applications across multiple tissue types, including skin, bone, neural, liver, gastrointestinal, cardiovascular, and dental tissues, using both natural and engineered PDVLNs in various disease models. Compared to mammalian exosomes, PDVLNs offer advantages such as reduced immune rejection and ethical concerns, enhancing their sustainability and appeal for regenerative medicine. However, challenges in clinical translation, including scalability, standardization, and safety remain. This paper consolidates current knowledge on PDVLNs, highlighting their versatility and providing insights into engineering strategies to optimize efficacy, ultimately outlining future research directions to advance their clinical potential. Plant vesicle-like nanoparticles (PDVLNs) may become a new avenue for the treatment of tissue injury, promoting tissue repair and regeneration through their intrinsic bioactivity or as drug delivery carriers. In addition, PDVLNs can be engineered and modified to achieve better results.

## 1. Introduction

Human tissue is the basic structural unit of the body. Its complex structure and function are coordinated to maintain normal physiological function, and different types of tissues work in concert to ensure effective organ and system functioning [1]. However, external trauma, disease, or congenital defects can cause tissue damage or defects in the human body, affecting the health and function of the whole organism [2]. Effective treatment is essential for restoring the physiological function of tissues and organs [3]. Traditional treatments, including surgical repair, transplantation, and physical therapy, face limitations such as donor scarcity, immune rejection, and surgical complications [3,4]. Stem cell therapy promotes regeneration and repair of damaged tissues by implanting pluripotent stem cells or inducing cell differentiation [5,6]. Nevertheless, unregulated proliferation and aberrant differentiation of stem cells could result in unpredictable therapeutic outcomes or tumor formation [7]. To reduce risk, cell-free therapies have become alternative therapeutic strategies [8], which utilize cell-secreted biomolecules to promote tissue repair and regeneration without directly using the cells themselves [9,10]. Extensive research conducted on mammalian-originated exosomes in the context of tissue injury repair highlights their pivotal role in cell-free therapeutic strategies. However, potential risks are associated with exosomes of mammalian origin, such as immune reactions and disease transmission [11].

Plant-derived vesicle-like nanoparticles (PDVLNs) are produced by plant cells through specific biological pathways and are often involved in plant cell–environment interactions, such as plant growth, development, and fighting pathogenic microorganisms [12]. PDVLNs harbor a plethora of biologically active molecules alongside plant-specific growth factors and hormones. These constituents confer diverse functionalities to PDVLNs, including antioxidant, immunomodulatory, and cellular proliferative properties, enabling their participation in and regulation of a variety of physiological processes [13]. Compared with mammalian exosomes, PDVLNs have a lower risk of immunogenicity and immune rejection [14]. Furthermore, PDVLNs circumvent ethical concerns associated with animal experimentation or embryo donation, rendering them more sustainable and socially acceptable. As their use in regenerative medicine evolves, numerous studies have shown their significant potential in areas such as tissue engineering, drug delivery, and disease therapeutics [15,16,17].

This review consolidates PDVLNs’ mechanisms, applications, and engineering potential, critically evaluating their advantages over existing regenerative agents and outlining a roadmap for overcoming current limitations. The literature was gathered through a comprehensive search of PubMed, Web of Science, and Google Scholar, using terms such as “plant-derived vesicle-like nanoparticles”, “tissue repair”, “regenerative medicine”, “plant exosome-like”, “nanovesicles” and related combinations to ensure a broad and relevant evidence base.

## 2. Overview of PDVLNs

### 2.1. Biogenesis Mechanism of PDVLNs

**Figure 1:**
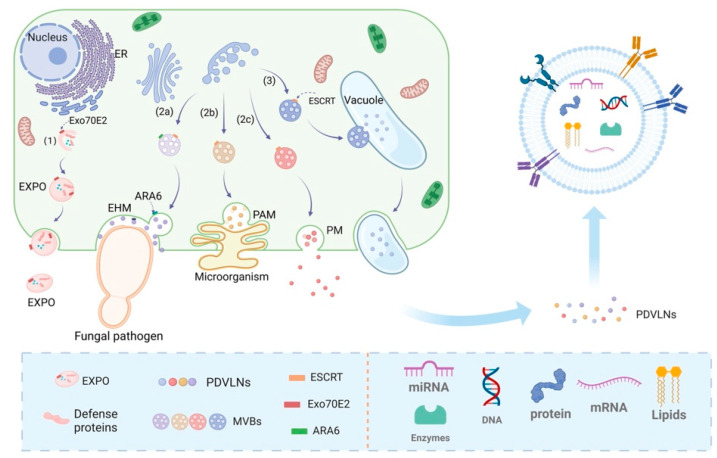
Biological pathways and structures of PDVLNs. Three pathways generate PDVLNs: (1) EXPOs merge with the PM, releasing vesicles to the cell wall. (2) MVB matures to form ILVs, aided by (2a) fungal infection, (2b) plant–microbe symbiosis, or (2c) direct fusion with the PM, releasing ILVs as PDVLNs. (3) MVBs fuse with vacuoles, which then fuse with the PM, releasing ILVs. Created in Biorender. Wang, Q. (2025) https://BioRender.com/wmt01v9.

PDVLNs were initially identified in carrot cells through electron microscopy (EM) during the 1960s [18]. Despite numerous successful studies on the isolation and characterization of PDVLNs, their biogenesis remains poorly understood [19]. Three potential biogenesis pathways include the multivesicular body (MVB), EXPO, and vesicular pathways [16]. The three biogenesis pathways are shown in Figure 1.

MVBs are late nuclear endosomes that arise from the formation of trans-Golgi networks (TGNs) or early endosomes [20]. These networks or early endosomes further facilitate the maturation of MVBs, leading to the formation of characteristic intraluminal vesicles (ILVs) within them, which are enriched with biologically active molecules such as RNA, lipids, and proteins [16]. The subsequent fusion of MVBs with the plasma membrane (PM) leads to exocytosis of ILVs into the extracellular space, eliminating intracellular waste products and enabling efficient delivery of bioactive molecules [21]. The MVB pathway, as a core mechanism, exhibits inherent diversity under a wide range of conditions [22]. Initially, during plant–microbe symbiosis, the fusion of MVBs with host-derived plexiform membranes (PAMs) is facilitated [23]. PAM in continuity with the PM releases ILVs into the clumped peripheral region between plants and fungi, potentially modulating disease resistance signaling and the inhibition of pathogenic microorganisms [24], as evidenced in *Arabidopsis* infected with turnip mosaic virus and in radish mosaic virus infection [25,26]. Second, MVBs mediate antifungal defense responses by fusing with membrane structures at the site of fungal infection [27]. In barley powdery mildew fungal infestation, MVBs may be released both by fusion with the PM and by secretion to specific sites of infection to release exosomes. Ultimately, MVBs may release PDVLNs extracellularly through a mechanism involving direct fusion with the PM [28,29,30]. The regulatory mechanisms of the MVB pathway under various environmental conditions equip plants with crucial molecular strategies to cope with external environmental challenges and combat pathogenic microorganisms, culminating in the formation and release of PDVLNs [29].

Recent advancements have elucidated the structural and functional properties of vesicles, showing that they contain ILVs and that specific MVBs can deliver ILVs to vesicles through a fusion mechanism with the vesicles [31]. Ultimately, upon the fusion of vesicles with the cytoplasmic membrane, the enclosed ILVs are extruded from the cell and enriched with sRNAs and proteins implicated in defense responses [32]. Under conditions of bacterial infection, MVBs are translocated to the central vesicle and degraded via endocytosed vesicles [33]. Concurrently, parietal cells utilize their membrane architecture to merge with the cytoplasmic membrane, enabling the secretion of vesicles into the extracellular milieu [26]. For example, the structure of MVBs with ILVs was observed in the epidermal cells of grapefruit in their PM and in the cytoplasm surrounding the central vesicle [34]. This is an important defense mechanism for plants against pathogen invasion and a pathway for the formation of released PDVLNs.

In *Arabidopsis thaliana* and tobacco cells, EXPO, an exocyst-positive organelle, aids in cytokinesis by facilitating the transfer of cytoplasmic contents to the cell wall, particularly during the fusion of PDVLNs with the cytoplasmic membrane [35]. The shape of EXPO is significantly different from MVB [36], with high-pressure frozen samples shown to have a spherical double-membrane structure [37]. The Exo70E2 protein in *Arabidopsis* plays a central role in EXPO formation [35]. This extracapsular protein is uniquely localized on discrete punctate structural domains of the cytoplasmic membrane and the double membrane architecture of EXPO, which is distinct from the inner membrane markers of the Golgi apparatus, TGN, and MVB [24]. During PDVLN release, EXPO fuses with the PM, liberating its inner single-membrane vesicle into the cell wall. Markers on both the inner surface of the vesicle and in its peripheral region indicate eventual rupture and release of Exo70E2 into the cell wall [26,37]. EXPO is not restricted to a specific cell type and is observed in diverse plant structures, and it represents a distinct form of secretion exclusive to plants [38]. Although the biological function of EXPO-mediated secretion of PDVLNs requires further exploration, this finding provides a completely new perspective for understanding the secretory pathway in plant cells.

PDVLN biogenesis has far-reaching implications for multifaceted scientific research. This study contributes to a deeper understanding of molecular interactions and regulatory networks within plant cells, uncovering the functions and roles of PDVLNs in a variety of biological processes. Moreover, this investigation provides new insights for the artificial preparation of PDVLNs by leveraging natural generation mechanisms and utilizing biological systems to drive their production. Upon successful synthesis, further analysis of their structure and properties can be conducted to facilitate clinical translation. Through an in-depth comparison with natural PDVLNs, the differences between artificially prepared PDVLNs and natural PDVLNs can be identified. This study is anticipated to provide pivotal technology for large-scale production and enable extensive applications of PDVLNs in various domains, including regenerative medicine.

### 2.2. Composition of PDVLNs

PDVLNs comprise lipids, nucleic acids, proteins, and other constituents [39]. Given their complexity and heterogeneity, there is no specialized database for PDVLN components [40]. This heterogeneity is reflected in two main aspects: (1) PDVLNs from different sources have different compositional components. (2) Vesicle-like nanoparticles (VLNs) from distinct parts of the same plant exhibit varying compositions. The biological functions of PDVLNs vary depending on preparation and isolation methods, highlighting the importance of investigating their composition.

#### 2.2.1. Lipids

Lipids play a pivotal role in the membrane structure of PDVLNs, influencing their formation, release, biological functionality, and cellular internalization [41]. Secretion of PDVLNs was reduced in *Arabidopsis* leaf cells lacking glycosylinositol phosphate ceramide (GIPC). In contrast, stimulation of *Arabidopsis* leaves with exogenous GIPC significantly increased PDVLN formation and release [42]. PDVLN lipids are mainly categorized into glycerolipids and phospholipids, which have significantly less cholesterol than animal cells do [43]. In grape-derived VLNs (GDVLNs), up to 98% of the lipids are phospholipids, with typical plant lipids, such as galactolipids, accounting for the remaining 2%, suggesting precise lipid sorting during Gp-DVLN biogenesis [44,45]. This precise lipid sorting was reflected in nanovesicles from different sources, such as the varying contents of phosphatidic acid (PA) and monogalactosylglycerol in VLNs of ginger or grape origin [46,47].

Notably, lipids are also active compounds in PDVLNs. In ginger-derived VLNs (Gg-DVLNs), denaturing proteins, removing RNA, or extracting lipids and reassembling them into liposomes inhibit the activity of NOD-like receptor protein 3 (NLRP3) inflammatory vesicles [48]. PAs in Gg-DVLNs, which make up 50% of phospholipids, interact with mammalian target of rapamycin (mTOR), an unconventional serine/threonine kinase pivotal in orchestrating the regulation of cell growth and proliferation [49]. Lipids also induce specific cellular responses in recipient cells, such as lipids within ginger-derived nanoparticles that upregulate *Foxa2* expression in intestinal epithelial cells, independent of RNA or protein influence [50]. Despite the anti-inflammatory and pro-cell proliferation functions of lipids, our understanding of them is limited; therefore, a more comprehensive lipidomic analysis of different PDVLNs is needed.

#### 2.2.2. Carbohydrates

PDVLNs are rich in protein, ranging from cellular communication to the delivery of bioactive substances, and their presence adds a new dimension to cell biology [51,52]. In conventional protein secretion, proteins originate from the endoplasmic reticulum, traverse the Golgi apparatus and TGN, and are ultimately secreted extracellularly. In unconventional protein secretion (UPS), however, proteins do not need to pass through these organelles, a pathway by which the UPS is formed in PDVLNs [53]. PDVLNs are also rich in membrane proteins that dominate and regulate communication between cells [54]. Moreover, the proteins on these vesicles contain specific markers, such as the PEN1 protein and TET8-GFP of PDVLNs in transgenic *Arabidopsis*, that reflect the origin and localization of the vesicles and the secretion mechanism [27]. Additional investigations are warranted to determine whether PEN1 or TET8 can function as universal protein biomarkers across diverse PDVLNs [55].

In addition to proteins that regulate cellular activity and function, the proteome of PDVLNs also includes enzymes related to carbohydrate and lipid metabolism and lattice protein chains and ATPases [56]. The presence of these proteins further corroborates the multifunctionality of PDVLNs [57]. More importantly, specific proteins in PDVLNs, such as water channel proteins, provide better stability to vesicles, enabling PDVLNs to be used as carriers for drug delivery [58]. Therefore, an in-depth understanding of the protein composition of PDVLNs reveals their biological importance and may also pave the way for their application in drug transportation in regeneration.

#### 2.2.3. Nucleic Acids

PDVLNs loaded with nucleic acids also show specificity, with constituent RNAs, especially miRNAs, of particular interest. PDVLNs exhibit a selective packaging mechanism for RNAs, with specific miRNAs and siRNAs preferentially loaded into PDVLNs. This is evident from the distinct RNA profiles observed between plant tissues and PDVLNs [59]. For example, the types and amounts of miRNAs in strawberry- and ginger-derived PDVLNs differ significantly from those present in their parental tissues [60,61]. The RNA composition of various plants may vary depending on the species, as demonstrated by the total RNA content extracted from fruit tissues or fruit-derived PDVLNs, such as grapefruit and grape, being significantly lower than that extracted from vegetable tissues or vegetable-derived PDVLNs, such as carrot and ginger [30]. This selective RNA packaging reveals the diverse biological activities of PDVLNs and the importance of RNA delivery in intercellular communication.

miRNAs perform a diverse array of regulatory functions within cells. They can bind to target mRNAs, thereby degrading them or inhibiting their translation [62]. PDVLNs provide a platform for carrying and delivering these functional miRNAs. For instance, Ginseng-derived VLNs (Gs-DVLNs) delivered 20 miRNAs to bone marrow mesenchymal stem cells (BMSCs), modulating genes linked to neural differentiation, maturation, and function. Both the GO and KEGG analyses underscore the significant potential of miRNAs within Gs-DVLNs to regulate neurally relevant processes [63]. Moreover, honey-derived VLNs efficiently suppressed NLRP3 inflammatory vesicle activity through *MiR-4057* [64]. Compared with animal miRNAs, plant miRNAs undergo natural 20-O-methylations, increasing their stability and protection against degradation and uridylation in various environments. These findings highlight the potential of plant miRNAs as drug carriers for therapeutic applications [65].

PDVLNs can serve as vehicles for RNA delivery, transferring RNA to mammalian cells to modulate gene expression, thus enabling plant–animal cellular communication. The RNA composition of PDVLNs varies among different plant species, necessitating further research to understand these differences and leverage them for the advancement of tissue repair and regeneration applications.

### 2.3. Preparation and Isolation Methods for PDVLNs

**Figure 2:**
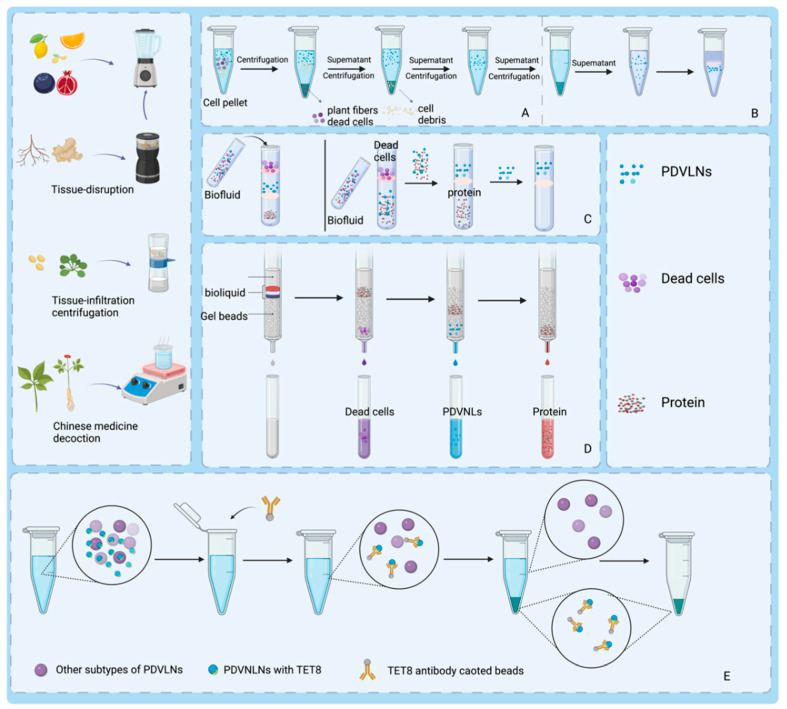
Different processes for obtaining PDVLNs. The preparation liquid is created via a blender, homogenizer, or tissue permeate along with traditional Chinese medicine decoctions. (**A**) Employs ultraspeed differential centrifugation, often with density gradient centrifugation (**B**), for PDVLN separation and purification. (**C**) Uses ultrafiltration to filter out organelles and dead cells, resulting in PDVLNs. (**D**) Employs SEC to separate PDVLNs on the basis of flow rates in a chromatographic column. (**E**) Utilizes immunoprecipitation with specific antibodies to isolate PDVLNs efficiently. Created in Biorender. Wang, Q. (2025) https://BioRender.com/5rmuegu.

The process of preparing PDVLNs begins with the extraction of sap from the plant material. The appropriate extraction method is chosen on the basis of the plant species. For juice-rich fruit plants such as grapes and apples, the tissue lysis method is used, which releases abundant juice by breaking the plant tissue cells and provides ideal raw materials for the subsequent preparation of PDVLNs. In contrast, for rhizomes such as ginseng, because of their denser tissues, the tissue osmosis method is used, in which the solvent gradually penetrates the interior of the plant tissues through osmosis to efficiently extract the desired sap. These two methods ensure efficient and accurate extraction of the raw materials required for the preparation of PDVLNs according to plant characteristics, laying a solid foundation for subsequent steps. In addition to the direct isolation of PDVLNs from fresh plants, boiled herbal tonics have been shown to be effective sources. Recent studies have demonstrated that herbal broths contain abundant lipids and substantial quantities of sRNA [66]. An in-depth analysis of the composition of the broth revealed that vesicular nanoparticles loaded with sRNAs exhibited antifibrotic and anti-inflammatory potential. These findings suggest that herbal tonics hold great promise for obtaining and isolating PDVLNs [67]. The main methods for the preparation and isolation of PDVLNs are shown in Figure 2.

Segregation involves the separation of PDVLN and non-PDVLN components and the separation of distinct types of PDVLNs [68]. Separation methods are fundamental to the study of nanoparticles and have an important impact on subsequent extensive preparation and characterization work. Various separation techniques may result in discrepancies in the size, shape, and surface characteristics of nanoparticles, consequently influencing their biological activity and applicability [69]. Consequently, exploring isolation techniques for PDVLNs is crucial for their potential applications in tissue repair and regeneration. The main methods for isolating PDVLNs and their respective advantages and limitations are summarized in Table 1.

In the preparation of PDVLNs, in addition to the selection of the separation method, the preparation conditions, including temperature, pH, centrifugation speed, and solution concentration, are equally crucial and significantly influence the characteristics and yield of PDVLNs [93]. Suresh et al. reported that PEG precipitation at pH values of 4 and 5 significantly increased the yield of PDVLNs [94]. The colloidal stability of VLNs of ginseng origin can be significantly enhanced by optimizing the preparation conditions and using a combinatorial approach when extracting the vesicles to obtain PDVLNs with the desired properties [95]. Therefore, the isolation method should be selected, and the preparation conditions should be optimized according to the plant materials and target properties to obtain high-quality PDVLNs samples. With the continuous development of nanotechnology, the purity and quality of nanoparticles are increasing. However, current isolation methods are unable to balance recovery and specificity. Therefore, new and efficient separation methods must be explored to achieve breakthroughs in the application of PDVLNs.

### 2.4. Characterization Methods for PDVLNs

The physical properties of PDVLNs are specific in two ways: 1. PDVLNs from different parts of the same plant may contain various particle subtypes with varying potentials. 2. Artificial factors, such as preparation and isolation methods and characterization instruments, may also lead to differences in the characterization of PDVLNs.

#### 2.4.1. Particle Size Distribution of PDVLNs

Particle size distribution is a critical parameter for characterizing PDVLNs, as it influences their stability, cellular uptake, and therapeutic efficacy in regenerative medicine. Techniques such as dynamic light scattering (DLS) and nanoparticle tracking analysis (NTA) are commonly used to measure the size of PDVLNs, providing insights into their heterogeneity and functional properties [96]. PDVLNs typically range from 30 to 200 nm in diameter, though this may vary depending on the plant source and isolation method [97].

#### 2.4.2. Morphology of PDVLNs

EM, a high-resolution imaging technique, is often used to explore the morphological features of PDVLNs. However, different imaging techniques can affect the morphology of PDVLNs; therefore, careful consideration is required when selecting an imaging method. Scanning electron microscopy (SEM) and transmission electron microscopy (TEM), two of the most prevalent EM techniques, require thorough sample fixation, dehydration, and staining, which may induce dehydration and deformation of vesicles, resulting in a cup-shaped morphology [98]. In low-temperature EM, PDVLNs typically exhibit a spherical morphology, suggesting that the cup-shaped structures observed via other EM techniques may result from dehydration. Additionally, EM enables precise characterization of PDVLN morphology and size and provides crucial insights into the mechanisms of their biogenesis. For example, Cai et al. observed the fusion of MVBs with the PM and the release of EVs via EM, supporting the presence of extracellular membrane structures in the plasma ectodomain [27].

#### 2.4.3. Potentiation of PDVLNs

DLS and NTA provide information on the surface charge state of PDVLNs [99]. The zeta potential is a metric for evaluating the stability of nanoparticle colloids. Typically, nanoparticles with zeta potential values between −30 mV and +30 mV exhibit enhanced stability [100]. PDVLNs exhibit diverse ranges of ζ-potentials, indicating their ability to exist independently without aggregation. Furthermore, the surface charge of PDVLNs varies, ranging from nearly neutral to approximately −50 mV, potentially attributable to variations in the components present in PDVLNs sourced from different plants.

#### 2.4.4. Surface Markers of PDVLNs

Identification of surface markers is critical for characterizing PDVLNs and confirming their utility in regenerative medicine. Western blotting and flow cytometry are two key techniques used to detect PDVLN surface markers, enabling precise validation and quantification.

Western blotting facilitates the detection of specific proteins on PDVLN surfaces. Western blotting of purified *Arabidopsis thaliana* PDVLNs, using commercially available mammalian exosomal kits, reveals the presence of surface marker tetraspanin proteins (CD9, CD63, and CD81) and endosomal sorting complexes required for transport (ESCRT)-associated proteins (TSG101 and ALIX), despite their plant origin [101]. These markers validate the vesicular identity of PDVLNs, although plant-specific proteins like PEN1 or TET8 may offer more targeted identification in certain contexts.

Flow cytometry offers high-throughput biomarker analysis. Flow cytometry represents a viable methodology for detecting biomarkers associated with PDVLNs, enabling high-speed multi-channel analysis with low sample concentration [102]. Optimized for nanoparticles (e.g., NanoFCM Nanoanalyzer), it quantifies PDVLN subtypes via fluorescent labeling, supporting therapeutic applications.

## 3. Mechanisms and Applications of PDVLNs in Tissue Repair and Regeneration

Tissue repair and regeneration are complex biological processes involving multiple cellular and molecular mechanisms. Plant-derived vesicle-like nanoparticles (PDVLNs) exhibit a range of biological functions—such as promoting cell proliferation, migration, differentiation, angiogenesis, immunomodulation, antioxidation, antiapoptosis, and microbiota regulation—that underpin their therapeutic potential across various disease models [103,104]. These functions facilitate intercellular communication, modulate recipient cell behavior, and create a microenvironment conducive to repair and regeneration [105,106]. Below, we systematically explore how these mechanisms translate into therapeutic applications, organized by specific tissue types and associated diseases.

### 3.1. Skin: Wound Healing and Facial Rejuvenation

Skin injuries, whether acute (e.g., trauma, burns) or chronic (e.g., diabetic ulcers), benefit from PDVLNs’ ability to regulate inflammation, promote cellular activity, and enhance vascularization. These nanoparticles promote cell proliferation, migration, and differentiation, key processes in skin repair [107]. For instance, aloe saponin-derived VLNs (AS-DVLNs) stimulate human dermal fibroblast (HDF) proliferation, while ginseng-derived VLNs (Gs-DVLNs) enhance proliferation of human keratin-forming (HaCaT) cells and human umbilical vein endothelial cells (HUVECs) [108,109]. Wheat-derived VLNs simultaneously promote proliferation of HDFs, HUVECs, and HaCaT cells, underscoring their biocompatibility and potential natural targeting mechanisms [110]. Cell migration, another critical step, is enhanced by Aloe vera peel-derived VLNs (Avp-DVLNs), which accelerate HaCaT and HDF movement to promote wound closure, and by Gs-DVLNs, which support HaCaT and HUVEC migration [108,110].

**Figure 3:**
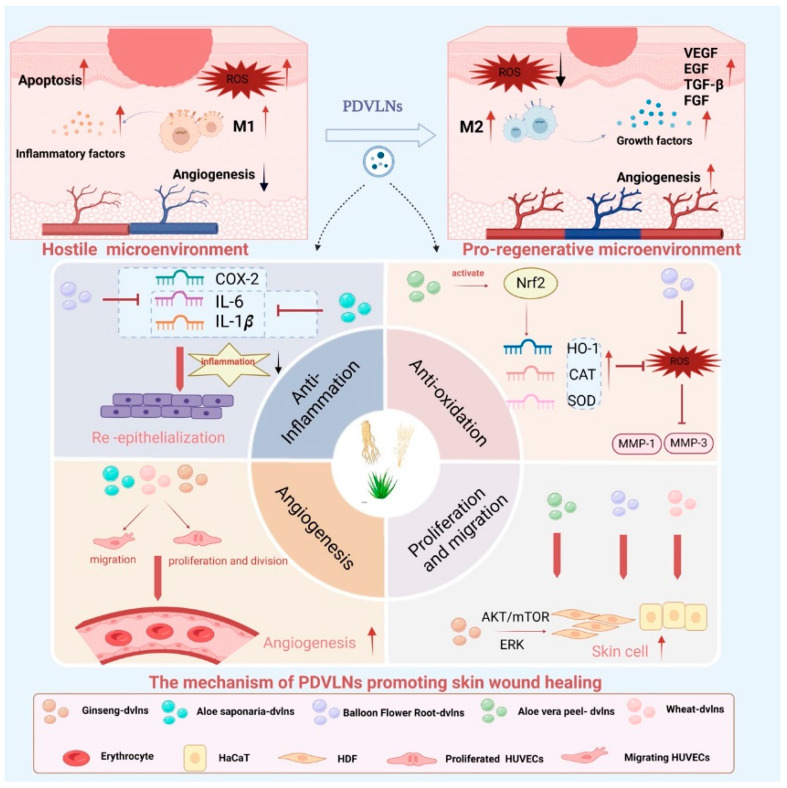
Promotion of wound healing by PDVLNs. In the hostile wound microenvironment, high levels of reactive oxygen species (ROS), pro-inflammatory M1 macrophages, apoptosis, and inflammatory factors (e.g., COX-2, IL-6, IL-1β) impede healing and angiogenesis. PDVLNs intervene by shifting the microenvironment towards a pro-regenerative state characterized by M2 macrophage polarization, increased growth factors (VEGF, EGF, TGF-β, FGF), and enhanced angiogenesis. The above panel illustrates four key therapeutic mechanisms of PDVLNs: (1) Anti-inflammation—inhibiting pro-inflammatory cytokines and promoting re-epithelialization; (2) Anti-oxidation—activating the Nrf2 signaling pathway and upregulating antioxidant enzymes (HO-1, CAT, SOD) to scavenge ROS and reduce MMP-1/3 expression; (3) Angiogenesis—facilitating migration, proliferation, and tube formation of endothelial cells; and (4) Proliferation and migration—enhancing proliferation and migration of skin cells through AKT/mTOR and ERK signaling pathways. Various PDVLNs from ginseng, aloe, balloon flower root, wheat, and other sources act synergistically to regulate wound healing processes. Created in Biorender. Wang, Q. (2025) https://BioRender.com/c4qwf70.

**Figure 4:**
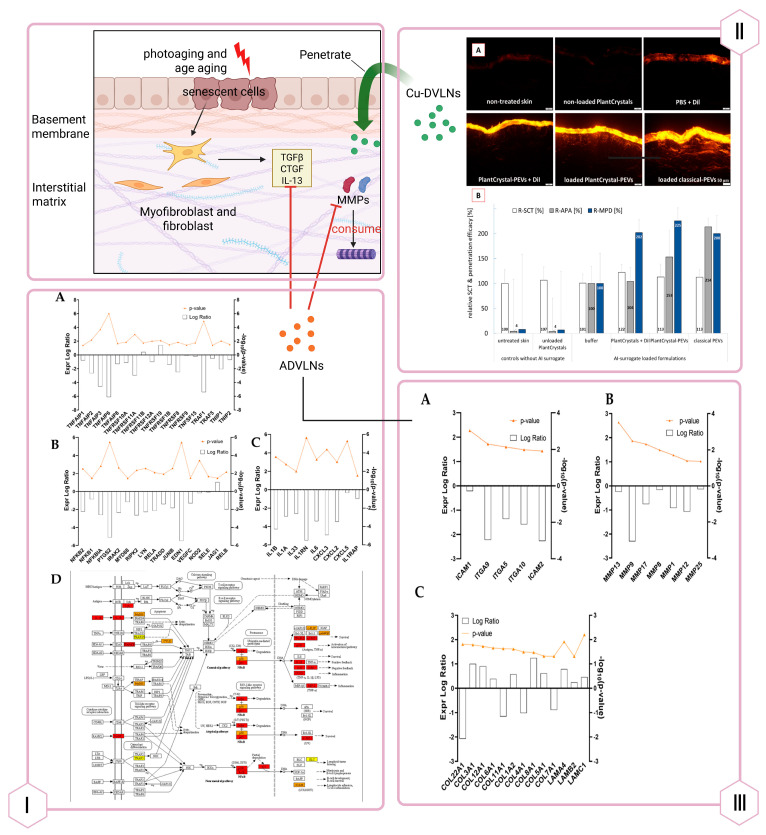
Antiaging Effects of PDVLNs. Panel I: ADVLNs exerted anti-inflammatory effects, downregulating NF-κB pathway genes (**A**,**B**) and IL-1 family factors/receptors (**C**), with KEGG analysis showing IL-1β/NF-κB pathway changes (**D**). Adapted with permission from Ref. [114]. Panel II: Cucumber-DVLNs (Cu-DVLNs) enhanced skin permeation, varying by formulation (**A**), and doubled penetration depth of lipophilic substitutes vs. buffer (**B**). Adapted with permission from Ref. [115]. Panel III: ADVLNs doubled collagen protein levels, reduced integrin/adhesion molecule expression threefold (**A**), decreased metalloproteinase expression (**B**), and doubled collagen chain expression (**C**). Adapted with permission from Ref. [114]. Created in Biorender. Wang, Q. (2025) https://BioRender.com/3hdbiqe.

Therapeutically, these functions translate into accelerated wound healing. In acute wounds, wheat- and ginseng-derived VLNs regulate HaCaT and HUVEC proliferation via ERK and Akt/mTOR pathways, attenuate inflammation, and promote angiogenesis in HUVECs, enhancing local microcirculation and nutrient delivery for re-epithelialization [109,110]. In chronic wounds, excessive matrix metalloproteinase (MMP) activity and reactive oxygen species (ROS) impede healing [111,112]. Grapefruit-derived VLNs (Gf-DVLNs) reduce oxidative stress in HaCaT cells, upregulate collagen type I and fibronectin expression, and promote proliferation and migration, expediting repair [112]. Avp-DVLNs activate the Nrf2 pathway, bolstering antioxidant defenses and enhancing migratory capacity, further supporting wound closure (Figure 3 and Figure 4) [113].

Beyond wound healing, PDVLNs address skin aging, characterized by chronic inflammation and extracellular matrix (ECM) degradation. Apple-derived VLNs (ADVLNs) downregulate the NF-κB pro-inflammatory pathway by inhibiting TLR4, reducing inflammation and increasing collagen synthesis (e.g., COL3A1, COL1A2) while suppressing MMPs (e.g., MMP1, MMP8), slowing ECM breakdown [114]. Wheat-derived VLNs boost type I collagen mRNA levels in fibroblasts, enhance proliferation and migration of endothelial, epithelial, and dermal cells, and suppress apoptosis, offering antiaging effects (see Figure 4) [109]. Unlike traditional antiaging products, PDVLNs’ natural composition minimizes allergic risks, and their small size enhances skin penetration, though further animal and clinical studies are needed [115].

### 3.2. Bone: Osteogenesis and Osteoporosis Treatment

Bone regeneration relies on cellular differentiation and proliferation, processes effectively promoted by PDVLNs. Yam-derived VLNs (YDVLNs) upregulate osteogenic markers such as osteopontin (OPN), alkaline phosphatase (ALP), and collagen type I (COL I) in ovariectomy-induced osteoporotic mice, facilitating bone regeneration [116]. Apple-derived VLNs (ADVLNs) enhance osteogenic gene and protein expression in MC3T3-E1 osteoblasts, supporting bone formation [117]. Tomato-derived VLNs increase chondrogenic markers and extracellular matrix (ECM) proteins in human adipose-derived mesenchymal stem cells (MSCs), outperforming lemon-derived VLNs and suggesting potential for cartilage repair in osteoarthritis [118].

**Figure 5:**
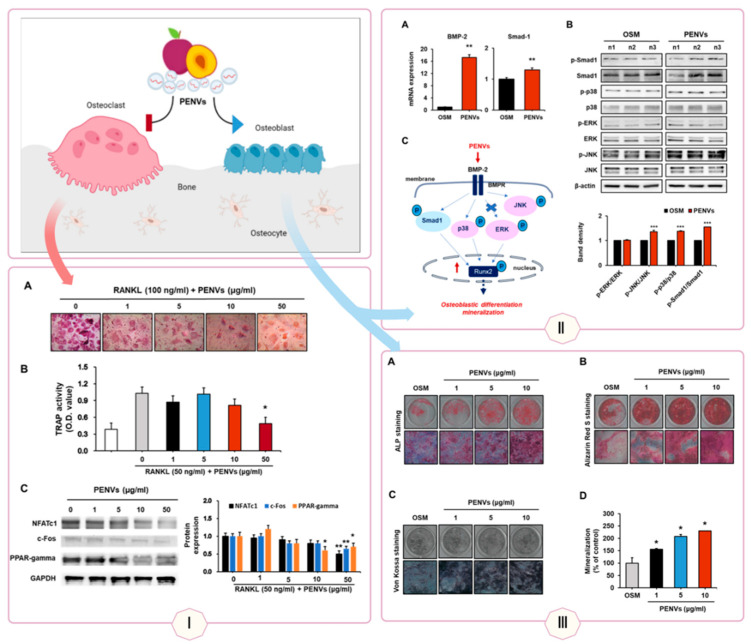
Promotion of Bone Regeneration by PDVLNs. PDVLNs exert multifaceted effects on bone regeneration by modulating osteoclasts and osteoblasts: Panel I: PDVLNs inhibit RANKL-induced osteoclastogenesis in RAW264.7 cells, evidenced by reduced TRAP-positive cells and enzymatic activity, indicating suppression of bone resorption. (**A**) TRAP staining and (**B**) TRAP activity (absorbance at 405 nm) was examined.Data presented as the means ± SD (n = 3). (**C**) Levels of the osteoclastogenesis proteins were estimated (n = 3). * *p* < 0.05, ** *p* < 0.01 between PENVs and control group (OSM; osteogenic medium). Panel II: In hBMSCs, PDVLNs downregulate pro-inflammatory genes (IL-6, IL-1β) while upregulating angiogenic markers (VEGF, MMP-2) at days 7 and 21, highlighting their dual anti-inflammatory and pro-angiogenic roles. (**A**) mRNA levels of BMP-2 and Smad-1 using Real-time quantitative polymerase chain reaction (RT qPCR). (**B**) Representative Western blot bands of different proteins are shown. (**C**) A schematic illustration depicting the effects of PENVs in promoting osteoblast differentiation. Data presented as means ± SD (n = 3). ** *p* < 0.01, *** *p* < 0.001 between PENVs and control group (OSM; osteogenic medium). Panel III: PDVLNs enhance osteogenic differentiation in hBMSCs, as shown by increased ALP staining/activity and elevated expression of osteogenic genes (ALP, RUNX2, OCN) at days 7 and 21. (**A**–**C**) Representative images of ALP staining, Alizarin Red S staining, and von Kossa staining are shown. (**D**) Ca deposition in extracellular matrix was quantified using Alizarin Red S dye. Data presented as the means ± SD (n = 3). * *p* < 0.05 between PENVs and control group (OSM; osteogenic medium). Adapted with permission from Ref. [120]. Created in Biorender. Wang, Q. (2025) https://BioRender.com/qjr4yh7.

Therapeutically, these mechanisms translate into treatments for bone-related disorders. YDVLNs’ induction of osteogenic differentiation supports their use in osteoporosis, restoring bone density in preclinical models (see Figure 5) [116]. ADVLNs’ effects on osteoblasts highlight their potential in fracture repair, while tomato-derived VLNs’ chondrogenic promotion offers a novel approach to osteoarthritis, addressing a condition with limited regenerative therapies. Additionally, Pueraria lobata-derived VLNs rebalance intestinal microbiota by metabolizing trimethylamine-N-oxide (TMAO), enhancing osteoblast differentiation and mineralization to alleviate osteoporosis, demonstrating a microbiota-mediated repair mechanism [119].

### 3.3. Nervous System: Neural Differentiation and Neuroprotection

Neural repair requires differentiation and protection against oxidative stress, both facilitated by PDVLNs [121]. Ginseng-derived VLNs (Gs-DVLNs) are internalized by bone marrow mesenchymal stem cells (BMSCs) in a time-dependent manner, delivering miRNAs enriched in neural-related signaling pathway genes [122]. This induces neural differentiation, evidenced by electrophysiological properties and neuron-like synaptic extensions, potentially via PI3K and transcriptional pathways, offering a novel strategy for stem cell-directed neural repair.

In neurodegenerative contexts, PDVLNs’ antioxidant and anti-inflammatory properties are critical. Blueberry-derived VLNs (Bb-DVLNs) inhibit ROS production and apoptosis in rotenone-treated HepG2 cells, a model relevant to Parkinson’s disease, where oxidative stress drives dopaminergic neuron loss [123]. Carrot-derived VLNs, rich in antioxidants, show promise for treating Parkinson’s and myocardial infarction by mitigating oxidative damage [124]. Gs-DVLNs’ miRNA delivery further supports their potential in neural regeneration, though specific uptake mechanisms by neural cells remain unclear [122].

### 3.4. Liver: Anti-Inflammatory and Antioxidant Effects

Liver repair benefits from PDVLNs’ immunomodulatory, anti-inflammatory, and antioxidant functions. Shiitake mushroom-, garlic chive-, and honey-derived VLNs inhibit NLRP3 inflammasome activation, reducing inflammation and promoting liver tissue repair [64,125,126]. Ginger-derived VLNs (Gg-DVLNs) similarly suppress NLRP3 activity, laying a foundation for treating Alzheimer’s and type 2 diabetes, conditions linked to liver dysfunction. Antioxidant effects are evident in Bb-DVLNs and Gg-DVLNs, which regulate Nrf2 distribution to mitigate hepatocyte apoptosis [127].

Therapeutically, these properties address liver injury. Lemon-derived VLNs enhance Lactobacillus rhamnosus GG (LGG) bile tolerance, upregulating LGG to support liver repair, demonstrating microbiota regulation’s role in regeneration [128]. Gg-DVLNs’ anti-inflammatory and antioxidant effects further position them as candidates for liver disease management, though optimal dosing requires further study.

### 3.5. Gastrointestinal Tract: Inflammation Control and Mucosal Repair

In the gastrointestinal (GI) tract, PDVLNs regulate inflammation and microbiota to promote repair. Ginger-derived VLNs (Gg-DVLNs) suppress NLRP3 inflammasome activity, reducing inflammation linked to inflammatory bowel disease (IBD) [129]. Blueberry-derived VLNs (Bb-DVLNs) modulate NF-κB and TLR4 expression in Caco-1 cells, downregulating IL-8β and IL-5 to mitigate intestinal inflammation [130]. Gg-DVLNs also increase probiotic Lactobacillus rhamnosus GG (LGG13A) populations, elevating IL-22 via the aryl hydrocarbon receptor (AHR) pathway to enhance antimicrobial immunity and mucosal repair at barrier surfaces [46].

**Figure 6:**
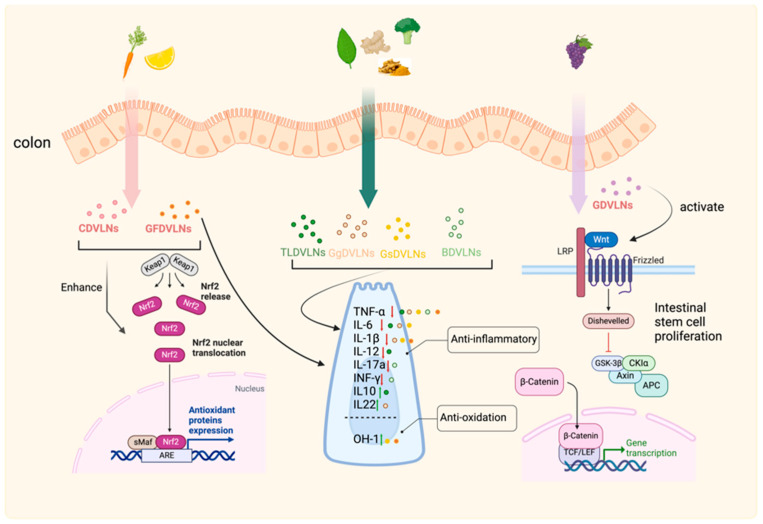
PDVLNs in Gastrointestinal Repair. Gg-DVLNs suppress NLRP3 inflammasome activity and enhance LGG13A/IL-22 levels in IBD models, while Bb-DVLNs reduce IL-8β and IL-5 via NF-κB/TLR4 modulation in Caco-1 cells, promoting mucosal repair. Created in Biorender. Wang, Q. (2025) https://BioRender.com/5r0gemp.

Therapeutically, these mechanisms support GI repair (see Figure 6). Gg-DVLNs’ anti-inflammatory and microbiota-modulating effects offer potential for IBD treatment, while Bb-DVLNs’ regulatory actions suggest broader applications in intestinal inflammation. By addressing dysbiosis-related damage, PDVLNs provide a promising approach to mucosal regeneration, though further studies on dosing and specificity are needed.

### 3.6. Cardiovascular System: Antioxidant and Angiogenic Support

Cardiovascular repair hinges on angiogenesis and oxidative stress mitigation, both enhanced by PDVLNs. AS-DVLNs promote angiogenesis in HUVECs, supporting nutrient delivery in skin wound healing, a mechanism applicable to ischemic tissues [108,131]. Carrot-derived VLNs’ antioxidant capacity counters myocardial hypoxia/reoxygenation-induced oxidative stress, protecting against cardiac hypertrophy and heart failure [124]. CDVLNs inhibit Nrf2 reduction in cardiomyocytes, further safeguarding against oxidative damage.

Therapeutically, these effects position PDVLNs as candidates for cardiovascular diseases. Carrot-derived VLNs’ high antioxidant yield suggests potential in myocardial infarction treatment, while their angiogenic properties could enhance revascularization in ischemic conditions, warranting further preclinical validation.

### 3.7. Dental Tissue: Periodontal Repair

PDVLNs contribute to periodontal repair through anti-inflammatory and microbiota-regulating functions, addressing damage in oral tissues. Ginger-derived VLNs (Gg-DVLNs) specifically target *Porphyromonas gingivalis*, suppressing virulence factor expression, which reduces inflammation and bone loss in periodontal tissues. Their immunomodulatory effects, such as decreasing pro-inflammatory cytokines (e.g., IL-6, IL-7) and increasing anti-inflammatory IL-10, further support tissue repair [132,133]. Additionally, Gg-DVLNs enhance probiotic populations like LGG13A, promoting a balanced oral microbiota conducive to regeneration [134].

Gg-DVLNs inhibit *Porphyromonas gingivalis* virulence, reducing inflammation and bone loss, while enhancing IL-10 and LGG13A levels to support periodontal tissue repair.

**Figure 7:**
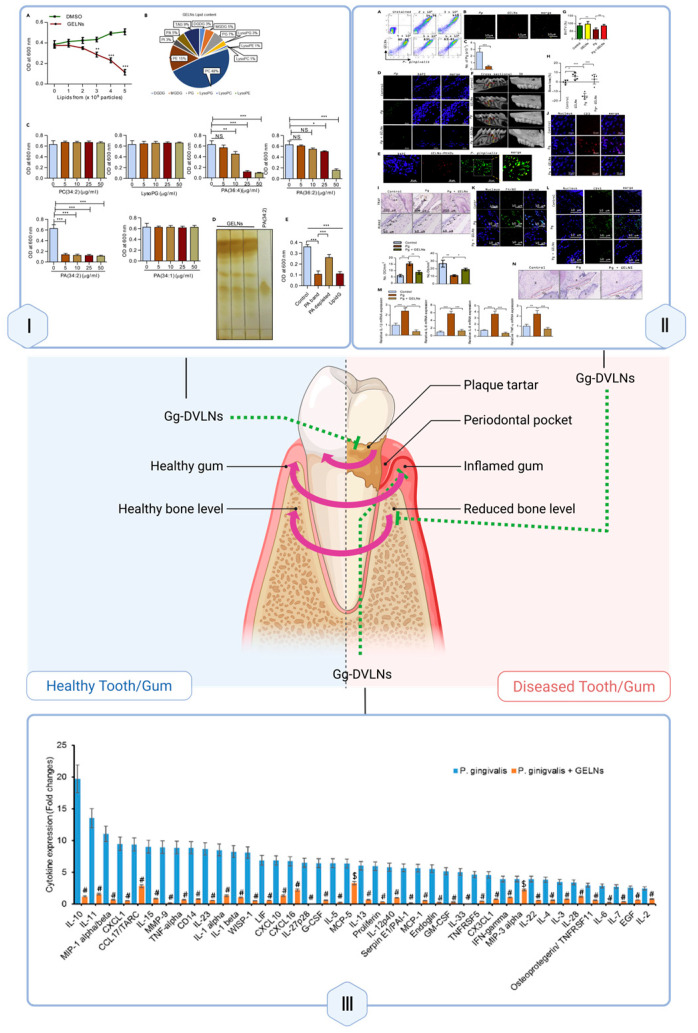
PDVLNs in Periodontal Repair. GELNs ameliorate periodontal disease by modulating host inflammatory responses and tissue repair processes. Panel I: In a *P. gingivalis*-infected periodontal murine model, GELNs are internalized by gingival tissue cells, attenuate alveolar bone loss, and modulate immune cell infiltration, as shown by histological staining and flow cytometry analyses. Panel II: Immunohistochemical and fluorescence microscopy images demonstrate that GELN treatment reduces neutrophil and macrophage presence in inflamed gum tissue, lowers markers, and promotes gingival epithelial integrity and bone preservation. Panel III: Cytokine array analysis of plasma from control, *P. gingivalis*-infected, and *P. gingivalis* + GELN–treated mice shows that GELNs significantly decrease circulating pro-inflammatory cytokines induced by infection. Adapted with permission from Ref. [132]. Created in Biorender. Wang, Q. (2025) https://BioRender.com/uz3nc5z.

Therapeutically, Gg-DVLNs show promise in treating periodontal disease by reducing inflammation and supporting alveolar bone preservation (Figure 7). Their ability to mitigate oxidative stress and regulate microbial dysbiosis positions them as a novel alternative to conventional therapies, though clinical validation is pending.

## 4. Therapeutic Advantages and Comparisons of PDVLNs in Regenerative Medicine

Plant-derived vesicle-like nanoparticles (PDVLNs) offer a compelling alternative to conventional regenerative therapies due to their unique biological and practical advantages [135]. Unlike stem cell therapies, which carry risks of tumorigenesis and immune rejection, PDVLNs provide a cell-free approach with low immunogenicity and minimal ethical concerns, stemming from their plant origin [136]. Their sustainability—bypassing animal-derived sources or embryo use—further enhances their appeal over mammalian exosomes, which, while effective in tissue repair, pose risks of disease transmission and immune activation [137]. For example, ginger-derived VLNs (Gg-DVLNs) suppress NLRP3 inflammasome activity without eliciting adverse responses, a feat less consistently achieved with mammalian counterparts [138].

Compared to synthetic nanoparticles, PDVLNs possess intrinsic bioactivity from their diverse cargos—lipids, proteins, and miRNAs—eliminating the need for extensive synthetic loading. Plant-derived compounds like quercetin or 6-gingerol exhibit pharmacological effects (e.g., antioxidant, neurogenic [139,140]) but suffer from poor solubility and bioavailability. PDVLNs overcome these barriers by encapsulating such bioactives within vesicular structures, as seen with apple-derived VLNs (ADVLNs) enhancing collagen synthesis in skin aging models [114]. Table 2 illustrates PDVLNs’ multifaceted roles across tissues—promoting angiogenesis in skin wounds, osteogenesis in bone defects, and neuroprotection in neural models—highlighting their superiority over single-target agents in addressing complex diseases.

PDVLNs exhibit distinct advantages over conventional regenerative therapies, including low immunogenicity, cost-effective scalability, and environmental sustainability, positioning them as viable alternatives to stem cell-based or synthetic nanoparticle approaches. Their regenerative efficacy varies by plant source, reflecting a remarkable versatility that enables tailored therapeutic applications, as evidenced in Table 2. In skin repair, aloe-derived PDVLNs bolster antioxidant defenses through Nrf2 pathway activation, while ginseng-derived PDVLNs enhance fibroblast and keratinocyte proliferation via ERK and Akt/mTOR signaling [110,141]. Wheat-derived PDVLNs further support wound closure by promoting cellular migration [109]. For bone regeneration, yam-derived PDVLNs upregulate osteogenic markers such as osteopontin and alkaline phosphatase in osteoporotic models [116], whereas tomato-derived PDVLNs drive chondrogenic differentiation, aiding cartilage repair [118]. In liver repair, shiitake mushroom-derived PDVLNs suppress NLRP3 inflammasome activity to mitigate inflammation [125], and blueberry-derived PDVLNs reduce oxidative stress through enhanced antioxidant enzyme expression [123]. Gastrointestinal repair benefits from ginger-derived PDVLNs, which inhibit NLRP3-mediated inflammation, and blueberry-derived PDVLNs, which modulate NF-κB and TLR4 pathways to alleviate inflammatory bowel conditions [144]. This plant-specific mechanistic diversity underscores the potential for selecting or combining PDVLNs to optimize tissue-specific regenerative outcomes, further amplifying their therapeutic promise. These attributes highlight PDVLNs’ capacity to address diverse clinical needs while overcoming limitations of traditional regenerative strategies. However, their full potential hinges on overcoming limitations in standardization and mechanistic understanding, as explored in subsequent sections.

## 5. Engineering PDVLNs for Enhanced Tissue Repair and Regeneration

To maximize PDVLNs’ regenerative potential, engineering strategies can enhance their stability, specificity, and delivery efficiency, building on their natural advantages. Surface modification, inspired by mammalian exosome engineering, can improve targeting precision [159]. For instance, conjugating PDVLNs with antibodies against periodontal pathogens like *Porphyromonas gingivalis* (Figure 7) could amplify Gg-DVLNs’ efficacy in dental repair (Table 2) [132,133,134]. Lipid reconstitution—reassembling PDVLNs with synthetic lipids—enhances membrane stability and uptake, as demonstrated by ginger-derived liposomes retaining anti-inflammatory activity [49].

Cargo enrichment offers another avenue, enabling PDVLNs to deliver tailored therapeutics. Loading with miRNAs (e.g., miR-162a for osteogenesis [160] and miRNAs for intestinal tract [161]) or small molecules (e.g., quercetin [140]) could bolster effects like Gs-DVLNs’ neural differentiation in BMSCs [63]. Controlled release systems, such as encapsulation in hydrogels [162], could sustain delivery in chronic conditions like diabetic ulcers or osteoporosis, extending PDVLNs’ impact beyond natural kinetics. Bioinspired synthesis—mimicking biogenesis pathways like MVB fusion—promises scalable production, with reconstituted grape-derived VLNs maintaining bioactivity [45].

These approaches, while transformative, must preserve PDVLNs’ low immunogenicity and biocompatibility. Advances in nanotechnology and bioengineering, paired with deeper insights into uptake mechanisms, will be pivotal to translating engineered PDVLNs into clinical tools, addressing limitations outlined in the next section.

## 6. Challenges and Future Directions in PDVLN-Based Regenerative Medicine

Despite the promising potential of plant-derived vesicle-like nanoparticles (PDVLNs) in tissue repair and regeneration, several challenges must be addressed to facilitate their clinical translation. To date, the therapeutic efficacy of PDVLNs has been primarily demonstrated in in vitro and in vivo studies, with limited clinical experience. However, emerging clinical trials are beginning to bridge this gap, underscoring the potential of PDVLNs as sustainable and low-immunogenic therapeutic agents. For instance, clinical trial NCT01668849 has demonstrated the efficacy of grape-derived exosome-like nanoparticles (GELNs) in mitigating oral mucositis in head and neck cancer patients undergoing chemoradiotherapy, highlighting their role in inflammation modulation and tissue protection. Similarly, trial NCT04879810 is investigating plant-derived exosomes, potentially incorporating curcumin, for alleviating inflammatory bowel disease symptoms, directly relevant to gastrointestinal repair. Additionally, trial NCT01294072 explores the capacity of plant-derived exosomes (grape-derived) to deliver curcumin to colon cancer tissues, showcasing their potential as targeted drug delivery vehicles. Trial NCT03493984 evaluates ginger- and aloe-derived exosomes for treating polycystic ovary syndrome, suggesting their utility in regulating inflammation and endocrine functions. These early clinical studies indicate that PDVLNs are progressing toward clinical applications, leveraging their low immunogenicity and sustainability to address diverse therapeutic needs. Nevertheless, further clinical validation is essential to establish their safety, optimal dosing, and long-term efficacy.

A significant challenge lies in the incomplete understanding of PDVLN biogenesis and uptake mechanisms. The pathways governing their formation and interaction with target cells remain poorly elucidated, necessitating advanced multi-omics approaches to uncover their molecular underpinnings. Furthermore, the heterogeneity of PDVLNs across different plant sources complicates their standardization and scalability for clinical use. Developing standardized protocols for isolation, purification, and characterization is critical to ensure reproducibility and therapeutic consistency.

For developers, navigating the regulatory landscape is critical to advancing PDVLNs toward clinical use. Due to their bioactive cargos and nanoscale properties, PDVLNs are likely to be regulated as drugs or biologics by the U.S. Food and Drug Administration (FDA) under the Center for Drug Evaluation and Research (CDER) or Center for Biologics Evaluation and Research (CBER), or as Advanced Therapy Medicinal Products (ATMPs) by the European Medicines Agency (EMA) [97]. The FDA’s 2014 guidance on nanotechnology products and 2019 public safety notice on exosomes emphasize the need for Good Manufacturing Practice (GMP) compliance, rigorous safety assessments, and validation of biological activity. For instance, the trial (NCT01713640) evaluating curcumin-loaded plant-derived nanoparticles for inflammatory diseases provides a precedent for regulatory pathways. Developers should engage early with regulatory bodies to clarify classification, define critical quality attributes (CQAs), and establish safety and efficacy standards tailored to specific clinical indications. As clinical trials progress and international regulatory guidance evolves, these efforts will support the compliant development of PDVLNs for tissue repair and regenerative medicine.

Looking forward, the future of PDVLNs in regenerative medicine is promising but hinges on addressing these challenges. Large-scale, well-designed clinical trials are needed to validate their therapeutic potential across diverse applications, such as skin, bone, neural, hepatic, gastrointestinal, cardiovascular, and dental tissue repair. Additionally, integrating multi-omics data with advanced bioinformatics will deepen our understanding of PDVLN mechanisms, paving the way for personalized and precision medicine applications. By overcoming these hurdles, PDVLNs have the potential to revolutionize sustainable and effective therapeutic strategies for tissue repair and regeneration.

## Figures and Tables

**Table 1:** Isolation methods for the PDVLN.

Separation Method	Principle	Advantages	Drawbacks	Make Superior	Reference
Ultracentrifugation	Separation of particles with large differences in size and density	Simple and economical	Formation of proteins, exosome aggregates; expensive equipment and expertise required	Combining density gradient centrifugation to obtain purer nanoparticles	[70,71,72,73,74,75]
Dense differential velocity gradient centrifugation	Centrifugal settling or sedimentation equilibrium in an inert gradient medium results in the formation of different separation zones.	Improved separation purity	Time-consuming, requires expensive equipment and specialized knowledge	Combine with differential ultracentrifugation	[69,73,74,75,76]
Ultrafiltration	Separation of large and small molecules by pressure membranes	Does not affect exosome activity, economical	Filter membranes prone to clogging, affecting recycling rates	Ultrafiltration pre-concentration of samples combined with size exclusion chromatography	[77,78,79,80,81,82]
Size exclusion chromatography (SEC)	Based on particle size differences	High purity, high yield	Inability to selectively extract specific size exosomes	Combine with ultrafiltration pre-concentration	[83,84,85]
Asymmetric flow field flow separation	Field effects cause particles to move at different speeds in different channels	Wide range of separated nanoparticles	Inability to distinguish between differently shaped aggregates	Immunoaffinity capture after preliminary separation using asymmetric flow field flow separation method	[84,85,86]
Immunoaffinity capture method	Utilizes high affinity antigen–antibody binding	Highly selective	Requires specific antibodies or magnetic beads, may affect biological function	Combine with asymmetric flow field flow separation	[55,87,88,89,90,91,92]
PEG-based precipitation	Utilizes the intrinsic negative charge property on the surface of PDVLNs	Simultaneously removing impurities such as nucleic acids and proteins	Non-specificity, not suitable for proteins that are sensitive to the presence of PEG	Combine with electrophoresis and dialysis	[87]

**Table 2:** Role of PDVLNs in promoting tissue repair and regeneration in different tissue and disease models.

Tissue	Source	Method of Administration	In Vivo or In Vitro Models	Potential Mechanism	Key Findings	Main Ingredients	Reference
Skin	Aloe-saponaria	co-incubation	In vitro: HDFs, HUVECs	Proliferation and migration of HDFs, Angiogenesis of HUVECs, Angiogenesis of HUVECs, Antioxidant	Promotes chronic wounds healing	unknown	[141]
	Ginseng	co-incubation	In vitro: HaCaT, HUVECs, BJ	Enhanced cell migration and angiogenesis, Increased secretion of extracellular matrix proteins, Regulation of proliferation and inflammatory response via ERK and Akt/mTOR pathways	Promotes wound healing	ginsenoside	[110]
	Wheat	co-incubation	In vitro: HDFs, HUVECs, HaCaT	Enhancement of wound healing-related gene expression; activation of fibroblast function, coordination of vascularization process	Promotes wound healing	unknown	[109]
	BFR	co-incubation	In vitro: HDFs	Down-regulation of pro-inflammatory cytokines; Promotion of HDF proliferation and migration	Promotes w (a–b) GELNs are taken up by P. gingivalis in mouse oral cavities. (c–e) GELNs reduce bacterial abundance and tissue colonization. (f–h) Micro-CT shows alleviated bone loss. (i–l) Staining reveals decreased osteoclasts, increased osteoblasts, and reduced immune infiltration. (m) Inflammatory cytokine levels are downregulated. (n) Histology shows preserved periodontal structure. Data are shown as mean ± SD from three independent experiments. *p* < 0.05, *p* < 0.01, * *p* < 0.001 (one-way ANOVA with Tukey’s multiple comparison test). ound healing	unknown	[142]
	*Aloe vera* Peels	co-incubation	In vitro: HaCaT, HDF	Decreased levels of ROS within HaCaT cells, Increased migration capacity of cells, Increased expression of mRNA for Nrf2, HO-1, CAT, and SOD genes	Promotes wound healing	unknown	[113]
	Grapefruit	co-incubation	In vitro: HaCaT, HUVEC	Reduction in ROS levels in HaCaT cells, Enhancement of proliferation- and migration-related gene expression in HaCaT, Enhancement of HUVEC tube formation	Promotes chronic wound healing	unknown	[143]
	Apple	co-incubation	In vitro: HDF	Inhibition of TLR4 activity, Down-regulation of NF-κB pro-inflammatory pathway, Enhancement of collagen synthesis, Inhibition of metalloproteinase production	Anti-aging of the skin	unknown	[114]
Liver	Shiitake mushroom	none	In vivo: mouse model of acute liver injury (GaiN, LPS induced)	Inhibition of NLRP3 inflammatory vesicle activation, Decrease in IL-6 activity	Combating FHF; preventing GaIn/LPS-induced acute liver injury	unknown	[125]
	Ginger	oral	In vivo: mouse model of alcoholic liver disease	Activation of Nrf2, Triggering of hepatic detoxification/antioxidant gene expression, Inhibition of ROS production	Protective effect against alcoholic liver injury in mice	shogaol	[144]
	Garlic	Oral or intravenous administration	In vivo: mice with acute liver injury	Inhibition of NLRP3 inflammatory vesicle activation pathway, Reduction in cysteinyl asparagin-1 autocleavage, inhibition of cytokine release and pyroptosis cell death in primary macrophages	Attenuating inflammation in chemically induced acute liver injury	DLPC	[126]
	Honey	none	In vivo: mice with acute liver injury	Inhibition of NLRP3 inflammatory vesicle activation, Reduction in IL-1β, IL-3, IL-6 and TNFα levels	Amelioration of inflammation and liver injury in acute liver injury	*MiR-4057*	[65]
	Blueberry	intragastric administration	In vitro: HepG2 In vivo: male C57BL/6 mice (6–8 weeks)	Acceleration of Nrf2 translocation, Decrease in AST and ALT levels, Improvement of insulin resistance, Inhibition of FAS and ACC1 expression	Treatment of NAFLD	unknown	[123]
	Lemon	co-incubation	In vitro: LGG	Inhibition of Msp1 and Msp3 production by RNase P-mediated degradation of specific tRNAs, Improvement of LGG tolerance to bile, Increase in LGG percentage	Regulates LGG to promote liver tissue repair	unknown	[128]
Bone	Plum	co-incubation	In vitro: MC3T3-E1 cells, mouse bone marrow primary osteoblasts	Modulation of BMP-2/MAPK/Smad-1 pathway	Promotes osteoblast activation, Reduces osteoclast differentiation	unknown	[120]
	Ginseng	co-incubation	In vitro: bone marrow-derived osteoclasts	Inhibits RANKL-induced signaling pathways, Regulates osteoclast maturation genes, Inhibits osteoclast differentiation	Anti-osteoporosis	ginsenoside	[145]
	Yam	oral	In vivo: mouse model of osteoporosis	Activates BMP-2/p-p38-dependent Runx2 pathway, Increases bone differentiation markers.	Promotes bone regeneration, Promotes osteoblast differentiation and mineralization	unknown	[116]
	Apple	none	In vitro: MC3T3-E1	Regulates BMP3/Smad3 pathway, Activates ERK and JNK-related signaling, Promotes osteoblast growth and differentiation.	Anti-osteoporosis	unknown	[117]
	*Pueraria lobata*	co-incubation	In vitro: hBMSC In vivo: Ovariectomy (OVX)-induced osteoporosis in rats	Promotes hBMSC differentiation and mineralization, Degradation of TMAO promotes autophagy.	Promotes bone formation and reduces bone resorption.	unknown	[119]
Colon	Grapefruit	oral	In vivo: DSS-induced colitis model in mouse	Up-regulation of HO-1 expression, Inhibition of IL-1β and TNF-α production in intestinal macrophages	Anti-inflammatory, immune-modulatory, maintenance of intestinal macrophage homeostasis	unknown	[146]
	Ginger	oral	In vivo: colitis model in mouse	Increased survival and proliferation of IECs, Decreased pro-inflammatory cytokines, Increased anti-inflammatory cytokines	Prevents and protects against IBD	6-gingerol	[49]
	Ginger	oral	In vitro: *Lactobacillus rhamnosus* (LGG) In vivo: mouse model	Activation of the AHR pathway, Induction of IL-22 expression	LGG-mediated inhibition of colitis in mice	unknown	[147]
	Mulberry bark	oral	In vitro: MC38 and human Caco2 colon cancer cell lines In vivo: mouse colitis model	Promoting HSPA8-mediated activation of the AhR signaling pathway and anti-inflammatory via AhR-COPS8	Prevention of colitis in mice	HSPA8	[148]
	Grape	oral	In vivo: rat	Modulating stem cell microenvironment, Promotes proliferation of Lgr5+ intestinal stem cells	Prevention of colitis in rats	unknown	[149]
	Grape	oral	In vivo: DSS-induced mouse colitis model	Induction of intestinal stem cell growth factor gene expression through the Wnt/β-catenin signaling pathway, Promotes proliferation of Lgr5+ intestinal stem cells	Promotes self-renewal of intestinal epithelium and accelerates the recovery of intestinal structure	unknown	[150]
	Tea leaves	oral	In vivo: mouse colitis model	Reduces ROS production, Inhibits pro-inflammatory cytokines, and Increases macrophage secretion of anti-inflammatory cytokines	Prevention of colitis-related colon cancer, antioxidant	galactose (CH_2_O_6_)	[151]
	Broccoli	oral	In vivo: mouse colitis model	Inhibition of pro-inflammatory cytokines and activation of AMPK are involved in inhibiting DC pro-inflammatory factor activation	Preventing DC activation and inducing tolerance to DC	unknown	[152]
	Turmeric	oral	In vitro: primitive 264.7 murine macrophages, NCM 460 and HT-29 colonic epithelial cells In vivo: ICR mice	Inhibits pro-inflammatory cytokine expression, promotes antioxidant gene levels, regulates gut microbiota composition and abundance, and remodels the immune microenvironment	Anti-inflammatory, antioxidant, relieves symptoms of intestinal inflammation	unknown	[153]
	Carrot	oral	In vitro: intestinal macrophages and stem cells. In vivo: B6.Cg-Tg(BAT-lacZ)3Picc/J mice	Promotion of Nrf2 nuclear translocation, induction of anti-inflammatory cytokines, antioxidant and activation of Wnt signaling gene expression	Anti-inflammatory, antioxidant, maintains intestinal homeostasis	unknown	[31]
	Pomegranate	co-incubation	In vitro: THP-1, Caco-2 cells	Improved Caco-2 cell survival at 5 ug/mL	Anti-inflammatory, antioxidant, promotes healing of intestinal epithelium	unknown	[154]
Nervous system	Garlic	oral	In vivo: mouse high-fat diet model	Inhibition of cGAS/STING/IDO1/AHR inflammatory signaling cascade response	Alleviating brain inflammation and obesity in mice	PA (36:4)	[155]
	*Momordica charantia*	none	In vivo: MCAO model in male SD rats	Up-regulation of AKT/GSK3β signaling pathway, inhibition of MMP-9, attenuation of BBB injury, and inhibition of neuronal apoptosis	Improving neurologic dysfunction in cerebral ischemia–reperfusion injury	Possibly related to *miR5266*	[156]
Cardiovascular system	*M.* *charantia*	intraperitoneal injection	In vitro: H9C2 cardiomyocytes In vivo: thoracic-irradiated 5–6 week old BALB/c nude mice	Promotes cell proliferation, inhibits apoptosis, attenuates DNA damage, scavenges mitochondrial ROS, and regulates p-AKT/AKT and p-ERK/ERK ratios	Reduces myocardial damage and fibrosis and is potentially protective against radiation-induced heart disease	unknown	[157]
	*Citrus limon* (L.) Osbeck	sublingual administration	In vivo: healthy participants	Effect on waist circumference and LDL-C	Cardiometabolic risk factors for management of normolipidemic participants	unknown	[158]
	Carrot	co-incubation	In vitro: H2C5 cardiomyoblasts and SH-SY2Y cells	Inhibits decreased expression of antioxidant molecules, protects cells from oxidative stress, and inhibits ROS production and ROS-induced apoptosis	Novel drug candidates for treatment of myocardial infarction and Parkinson’s disease	unknown	[124]
Oral cavity	Ginger	oral	In vitro: *Porphyromonas gingivalis* surface In vivo: mouse model of chronic periodontitis	Acts with heme-binding protein 35 (HBP35) to inhibit the pathogenicity of *Porphyromonas gingivalis*, reduce the expression of anti-inflammatory factors in periodontal tissues, and reduce alveolar bone loss	Prevention/treatment of chronic periodontitis	PA (36:2)	[132]

## Data Availability

Not applicable.
